# Cases in Practice

**Published:** 1858-02

**Authors:** Charles Brackett

**Affiliations:** Rochester, Fulton County, Indiana


ARTICLE III.

### CASES IN PRACTICE.

BY CHARLES BRACKETT, M. D., ROCHESTER, FULTON COUNTY, INDIANA.

*Nov.* 21*st*, 1857.—I was called to see a case of what was considered paraphimosis, producing gangrene penis. The line of demarkation had already formed, and the corpus cavernosa *appeared*, with the skin, completely ulcerated through, only the corp. spongiosum remaining to be cut through. This line of ulcerative amputation was about half an inch from the pubis. My going out was to amputate; but as nature was doing the job so completely, I concluded to wait, and see how she would finish it.

The history of the case—very defective, of course—was this: Some eight or ten days previously, the boy, —— Scott, aged 12, complained of some lameness, but attended to the chores as usual till the 20th, when he suffered so much that his mother questioned him more closely, and he said he had pain in his bowels, but wanted nothing done. The mother compelled him to have the bowels fomented, and then discovered that the penis was completely black, and nearly off. She then sent for my partner, Dr. Gould, who supposed the case one of strangulation and gangrene from paraphimosis. The next day I saw him, and remarked that the only case I had ever seen similar was in the practice of Dr. White, (my preceptor,) of Cherry Valley, N. Y., and that was caused by a string having been tied about the member to prevent incontinence of urine. There the string was discernible, and easily removed. In this case the boy said he had never tied the member, or hurt it in any way. *I foolishly believed him*, and thought of the case as did Dr. Gould, very much against my own convictions, however, and left the case to nature till to-day, when I visited him again. Penis still black; ulceration *no deeper*. Why? I asked myself. Because a string must have cut through so deep, and will not cut any deeper. I took a probe, felt, and saw the string, a small, strong hemp one; raised it with small forceps, and cut it with scissors, much to the relief of the boy and myself. So much for *trusting to the story of a patient suffering from disease, or injury of any part of the genitalia, male or female.*

The result of the case remains for the future to disclose. Nature can and will do very much, if she only has a chance. Here is a boy, well formed and hearty, who must have suffered all the horrors of retention and strangulation for days, until the string had cut through the skin, part of corpora spongiosa and corpora cavernosa; after which the meatus became pervious, the pressure of the string being removed, and the retention, of course, ceased; yet the penis, only being attached by the canal of the urethra, was about to be amputated. He was perfectly silent, unwilling to confess his fault, though he lost his penis for it. Here is a good nut for your moral philosophers to crack.

